# Hotspot-targeted life support training reduces code blue alarms in a tertiary children’s hospital: a single-center observational before–after study

**DOI:** 10.1016/j.resplu.2026.101520

**Published:** 2026-10-06

**Authors:** Volker Nils Umlauf, Ruth Bieri, Daniela Studer, Thomas Riedel

**Affiliations:** a Division of Pediatric Intensive Care Medicine, Department of Pediatrics, Inselspital, Bern University Hospital, University of Bern, Freiburgstr. 15, 3010 Bern, Switzerland; b Pediatric Medical Emergency Team, Department of Pediatrics, Inselspital, Bern University Hospital, University of Bern, Freiburgstr. 15, 3010 Bern, Switzerland

## Abstract

**Background:**

Code Blue emergency alarms are activated by hospital staff for life- threatening patient situations. Despite their low frequency in children’s hospitals, ward staff must bridge the first critical minutes until arrival of dedicated emergency response teams. Thus, the aim of this study was to identify hotspots and causes of emergencies, and to evaluate whether a hotspot-specific pediatric basic life support training (PBLS) reduces Code Blue alarms.

**Methods:**

This analysis included all Code Blue alarms at the University Children’s Hospital Bern before (January 2021–April 2023) and after (January 2024–December 2025) introduction of a hotspot-targeted PBLS program. Following identification of ward-specific hotspots and recurring emergency situations during Period 1, standard PBLS training was reorganized and supplemented by short simulation-based trainings tailored to the most frequent local emergencies. Variables between periods were statistically compared. Incidence rates were calculated per 1000 patient days.

**Results:**

65 Code Blue alarms were analyzed. Respiratory emergencies and seizures were the most common causes, with infants and toddlers being the most affected age group. No weekend but a lunch-time effect was observed. Code Blue incidence decreased from 0.58 to 0.21 alarms per 1000 patient-days after implementation (IRR 0.36, 95% CI 0.19–0.66, *p* < 0.001). Fragility analysis confirmed result robustness, with no evidence of bias from missing data, case mix index, PICU transfer rates, or non-Code Blue Medical Emergency Team (MET) consultations.

**Conclusions:**

Hotspot-specific PBLS training was associated with a marked reduction in pediatric Code Blue alarms. Identification of local emergency patterns may help hospitals develop targeted ward-based training strategies to improve in-hospital emergency management.

## Introduction

Acute life-threatening events in pediatric wards are uncommon but often highly dynamic, requiring immediate intervention. Different in-hospital systems enable immediate mobilization of specialized emergency teams for such events.^1^, ^2^ “Code Blue” calls are most frequently initiated in children in an acute event or decompensation, particularly of respiratory, cardiopulmonary, or neurological nature. However, there is no standardized definition of a „Code Blue“, and its interpretation varies across institutions. Furthermore, Code Blue systems may overlap with Medical Emergency Teams (MET), which respond at the bedside to patients showing early signs of clinical worsening, aiming to prevent further deterioration.^3^, ^4^, ^5^

While some studies suggest benefits,^6^, ^7^ consistent evidence for mortality reduction,^7^, ^8^ pediatric intensive care unit (PICU) admission rate, cardiac arrest rate, or length of stay in pediatric MET systems remains lacking.^6^, ^9^, ^10^, ^11^ Nevertheless, simulation-based life support and team training programs improve recognition of patient deterioration, adherence to resuscitation protocols, and team performance in METs as well as in ward staff,^12^, ^13^, ^14^, ^15^, ^16^, ^17^, ^18^ though the direct impact on medical emergency team (MET) activation rates is less reported.

Addressing these gaps offers an opportunity for novel approaches that target improvement in in-hospital emergency management not only from the perspective of the emergency teams itself, but also through the frontline ward teams managing deteriorating patients first.

The aim of this study was to retrospectively systematically analyze all Code Blue alarms during the period before implementation of a hotspot-specific PBLS training program and to compare them with a second period following the introduction and continued delivery of this training. We hypothesized that this hotspot-targeted training would be associated with a reduction in the rate of Code Blue alarms.

In addition, we present our ward- and department-specific training concept in comparison to the previously only used basic life support courses and describe the additional structural measures implemented in parallel.

## Methods

### Study design and setting

This is a retrospective observational study including all documented Code Blue alarms of the University Children’s Hospital Bern between January 1, 2021, and April 30, 2023 (Period 1), and from January 1, 2024 to December 31, 2025 (Period 2). After identifying “hot spots” with recurring alarm causes, we introduced additional trainings at the sites, thematically focused on previous code blue alarm patterns combined with basic life support training (Table 1), but in their own environment and using their own equipment, which was adapted and unified for the whole hospital (Table 1). Activations from the trauma/resuscitation room at the Emergency Department were excluded.

**Table 1:** Comparison of PBLS course curriculum and emergency equipment before and after implementation.

	**Period 1: Pre-implementation**	**Implementation period and Period 2: Post-implementation**
**Course curriculum**		
PBLS	• New employees • Every 2 years • Central location	• New employees • Every 2 years • Central location
Extended PBLS	−	• Simulation based + call training + handover training • Once-twice a year • On units/wards/departments Special addressed training *Infant/cardiology ward:* recognition of the deteriorating cardiac patient *Haemato-oncology ward:* anaphylaxis *Pneumology/general pediatrics:* respiratory incidents, tracheostomy complications
Staff	Nurses	• Nurses • Ward/unit physicians • Medical emergency team

**Resuscitation trolley**		
*Airway/Breathing*	• manual resuscitation bag (multipurpose, neonatal/pediatric/adult) • face masks (6 sizes, oval and round forms) • Guedel airways (6 sizes) • Laryngoscope handles (2 sizes) • Blades (sizes 00–3, Miller/MacIntosh) • Endotracheal tubes (sizes 2.0–7.5, duplicate stock) • Suction catheters (Ch. 6–12) • Yancauer suction tip • Fixation material	• Manual resusctitation bag-valve mask set (disposable; pediatric/adult) • Non-rebreather oxygen mask with reservoir (pediatric) • Suction catheters (Ch. 6–12)
*Medications*	• Epinephrine (ampules) • Dobutamine (ampules) • Midazolame (ampules) • Ketamine (ampules) • Opioids (selected) • Normal saline 500 ml • Normal saline • Glucose-containing pediatric maintenance fluids	• Epinephrine (pre-filled syringes, 1:10.000) • (Epinephrine auto-injecor, only at hem-onc) • Ringer’s lactate • Normal saline (pre-filled syringes)
*Vascular Access Other material*	• Needles, syringes, adhesives, gauze sponges • IV catheters (20G-26G) • IV administration sets • Blood transfusion sets • Penlight	• Needles, syringes, adhesives, gauze sponges • IV catheters (20G–26G) • IV administration sets • Penlight

The Children’s Hospital Bern is a 133-bed tertiary care, university-affiliated center encompassing all pediatric subspecialties in an alone-standing building within the Inselspital University Hospital campus. Approximately 6600 inpatients, 50,000 outpatients, and 23,000 emergency patients are treated annually, including approximately 900 admissions to the 12-bed Pediatric Intensive Care Unit (PICU). Within the same building, 2 adult internal medicine outpatient units, the hospital’s central physiotherapy pool, and a restaurant with up to 900 daily visitors are also located. Approximately 320 staff members are present on a typical weekday.

During Period 1, institutional Pediatric Basic Life Support (PBLS) training was mandatory biennial for all clinical staff with direct patient contact. New employees were required to complete an initial training course within the first 2 days of tenure. Resuscitation trolleys on every unit were equipped with advanced life support materials (Table 1) and varied between some wards.

Following a comprehensive hotspot analysis, the training and equipment infrastructure underwent a 6-month reorganization (May 2023–December 2023): While centralized PBLS courses for new hires and mandatory biennial refreshers remained in place, the curriculum was expanded by ward-based PBLS sessions with short-cycle simulations tailored to unit-specific patient populations and emergency patterns identified during hotspot analysis. Training focused on early recognition of deterioration, ward-specific emergencies, structured Code Blue activation, maintenance of high-quality PBLS measures until MET arrival, and structured handover to the emergency team. During the 6-month reorganization period, all units, including nursing staff, ward physicians, and the MET, were trained at least once (Table 1).

Post-implementation (Period 2), simulation-based PBLS training sessions were maintained at 1–2 months intervals on a rotational basis across the various wards and units.

The second observation period was limited to 24 months due to an institutional database migration, that temporarily prevented further systematic documentation.

### Activation pathways and criteria

All staff members are authorized to trigger a Code Blue alarm by dialing 8888 from any internal phone. Calls are received by a dedicated PICU dispatcher nurse using a one-question protocol focused solely on emergency location to avoid delays and misinterpretation (“Children’s Hospital Emergency Call Center – where is the emergency location?”). Simultaneously, the entire emergency team is activated via pager and immediately proceeds to the emergency location. Activation criteria include:

• Any form of resuscitation
• Any subjectively perceived life-threatening deterioration
• Impending decompensation
• serious concerns about a person

Separately, staff can trigger a conventional MET call via a different pager when they are concerned about a deteriorating patient or require assessment, clinical advice, or discussion. The activation criteria were intentionally broad, with some overlap between Code Blue activation and direct MET consultation being accepted, following international MET recommendations.^12^ Patients are assessed at the bedside promptly, but no later than 30 min after activation. Both distinct pathways are managed by the same team and thus share the institutional designation “Pediatric Medical Emergency Team (“PedMET”)” in our hospital.

After stabilization, the responding and ward teams jointly determine the patient’s further management, monitoring requirements, and location of care. Depending on the patient’s condition, the responding team may transfer the patient to another unit, such as the PICU; the ward team may arrange a secondary transfer; or the patient may remain on the ward for continued care. Patients initially managed by ward staff without Code Blue activation may continue to receive care independently, although the MET remains available at any time for reassessment, clinical advice, or joint management decisions.

Redundant communication pathways were established to minimize the risk of missed emergency calls: Calls placed inadvertently to the adult hospital emergency number or the emergency department are received by the respective service and subsequently relayed via the Children’s Hospital emergency number, thereby activating the pediatric emergency team.

The team itself is based in the PICU and consists of a pediatric intensivist, a PICU fellow or resident, and a PICU nurse, carrying fully stocked emergency equipment for patients ranging from preterm infants to adults (Supplemental 1 Equipment), and is responsible for all individuals within the Children’s Hospital building, including patients, staff, visitors, and adult patients from extra-pediatric departments.

### Primary and secondary outcomes

The primary outcome was the incidence of Code Blue alarms per 1000 patient-days, comparing the pre-intervention and post-intervention periods. Secondary outcomes included the temporal, spatial, and clinical characteristics of Code Blue events, including patient age, and sex, indication, timing, location, interventions, response times, and patient disposition. The frequency of MET activations was additionally assessed as a measure of utilization of the team for non-Code Blue activations.

### Data collection

For each Code Blue event, the handwritten Code Blue protocol was systematically reviewed. The following variables were recorded and transcribed into an anonymized electronic database (Microsoft Excel, Microsoft Corporation): age, sex, date of alarm (including day of the week), response times (activation, arrival, time on scene, end of response), location, indication/cause, transfer location or stay at the scene.

To capture episodes where the response team was activated through the MET pathway without triggering a Code Blue alarm, we extracted data from the hospital’s internal billing records. These records include charge codes for MET-related consultative assessments requested by ward teams. Cases identified through internal billing were cross-checked with the Code Blue protocols to exclude duplicates (*n* = 2). Non-Code blue MET consultations were analyzed separately, not as a second study cohort, but as a complementary comparison measure of consultation frequency and workload beyond formal Code Blue activations.

### Statistical analysis

Statistical analyses were performed using R Software for Statistical Computing, version 4.5.3 (R Foundation for Statistical Computing, Vienna, Austria), within the RStudio integrated development environment, version 2026.04.0 (Build 526; Posit PBC, Boston, MA) and supplemental packages (Supplemental 1 R packages).

Continuous variables are presented as medians with interquartile ranges (IQR) and were compared using the Wilcoxon rank-sum test. Categorical variables are reported as frequencies and percentages. Differences in categorical variables between the two study periods were assessed using Fisher’s exact test.

Because of differing observation periods (28 vs. 24 months), Code Blue incidence rates per 1000 patient-days were compared using Poisson testing, reporting incidence rate ratios (IRR) with 95% confidence intervals (CI). Robustness of the primary outcome was assessed by sensitivity and tipping-point analyses evaluating the impact of potential underreporting in Period 2. In addition, the Case Mix Index^19^ was used as a benchmark between the two periods, based on the hospital's billing data.

Distribution of categorical variables across the cohort (e.g., age groups, weekdays, diagnoses, and activation locations) was analyzed using Chi-square goodness-of-fit testing with post-hoc standardized Pearson residuals (*z* > 1.96 considered significant). For analysis of a potential weekend effect, weekdays were grouped into “early week” (Monday–Thursday) and “extended weekend” (Friday–Sunday), applying weighted expected frequencies according to the 4:3 day ratio.

Temporal distribution across nursing shifts (day, evening, night) was analyzed using global and post-hoc Fisher’s exact tests with Bonferroni correction for multiple comparisons. Statistical significance was defined as *p* < 0.05.

### SRQR and ethical approval

The study was conducted and reported in accordance with the Standards for Reporting Qualitative Research (SRQR). As this study met the criteria for a quality control study, the Ethics Committee of the Cantone of Bern determined that – in accordance with the Swiss Human Research Act (HFG) – no further ethical review was necessary (BASEC-ID 01306).

## Results

### Activation frequency and incidence

Over the total 52-month study period, 65 Code Blue alarms were recorded. Period 1 accounted for 50 alarms, while Period 2 accounted for 15 activations. Incidence rates significantly decreased from 0.58 per 1000 patient-days in Period 1 to 0.21 per 1000 patient-days in Period 2 (Fig. 1), mainly due to a reduction of alarms during the evening shift (*p* < 0.001, Fig. 2). Notably, this reduction occurred despite slightly increasing clinical complexity (Case Mix Index CMI 1.41 vs. 1.44).

**Fig. 1:**
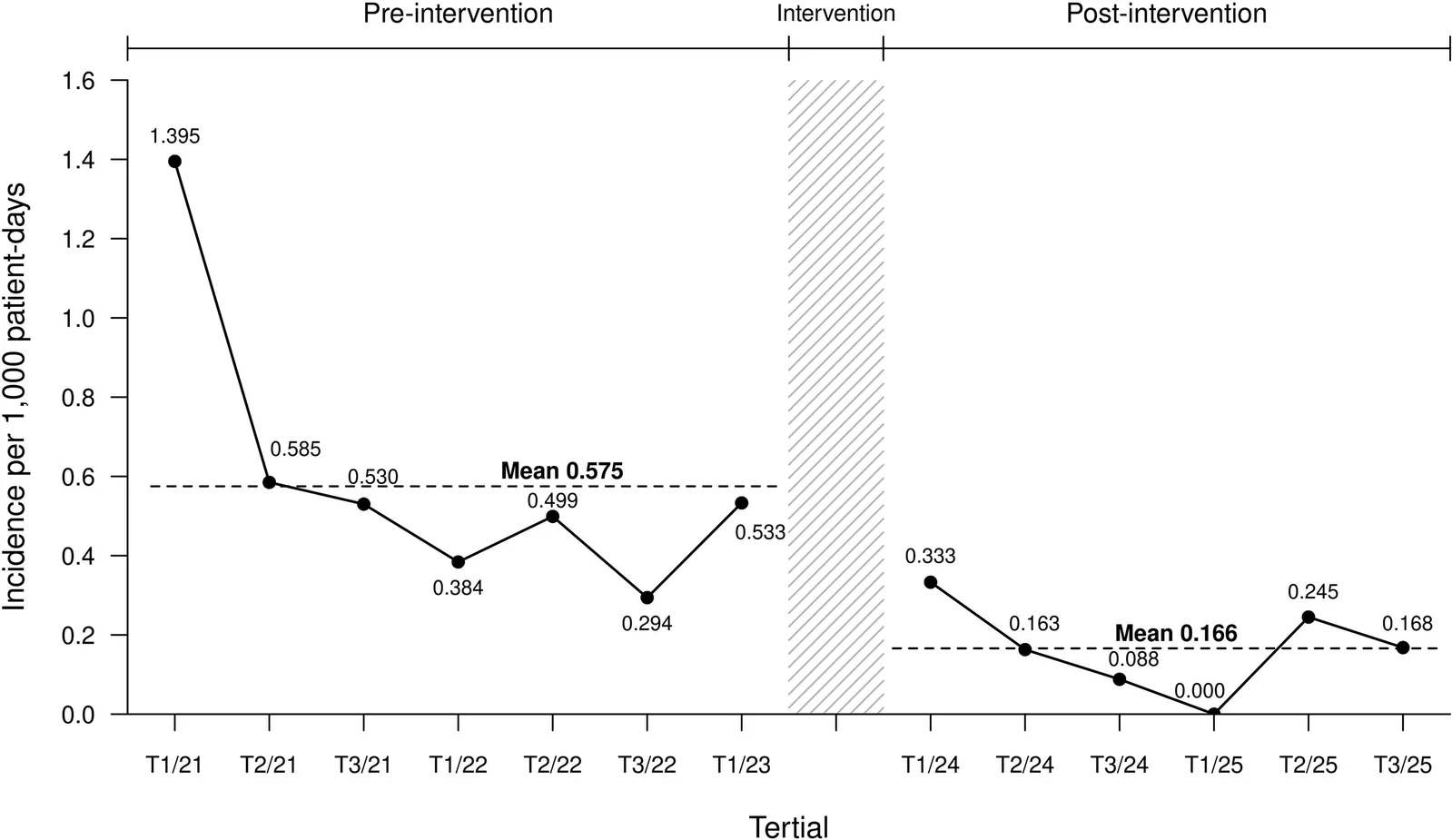
**Code Blue incidence rates per tertials; significant reduction of alarms in total between Period 1 and Period 2 (*p* = 0.00026)**.

**Fig. 2:**
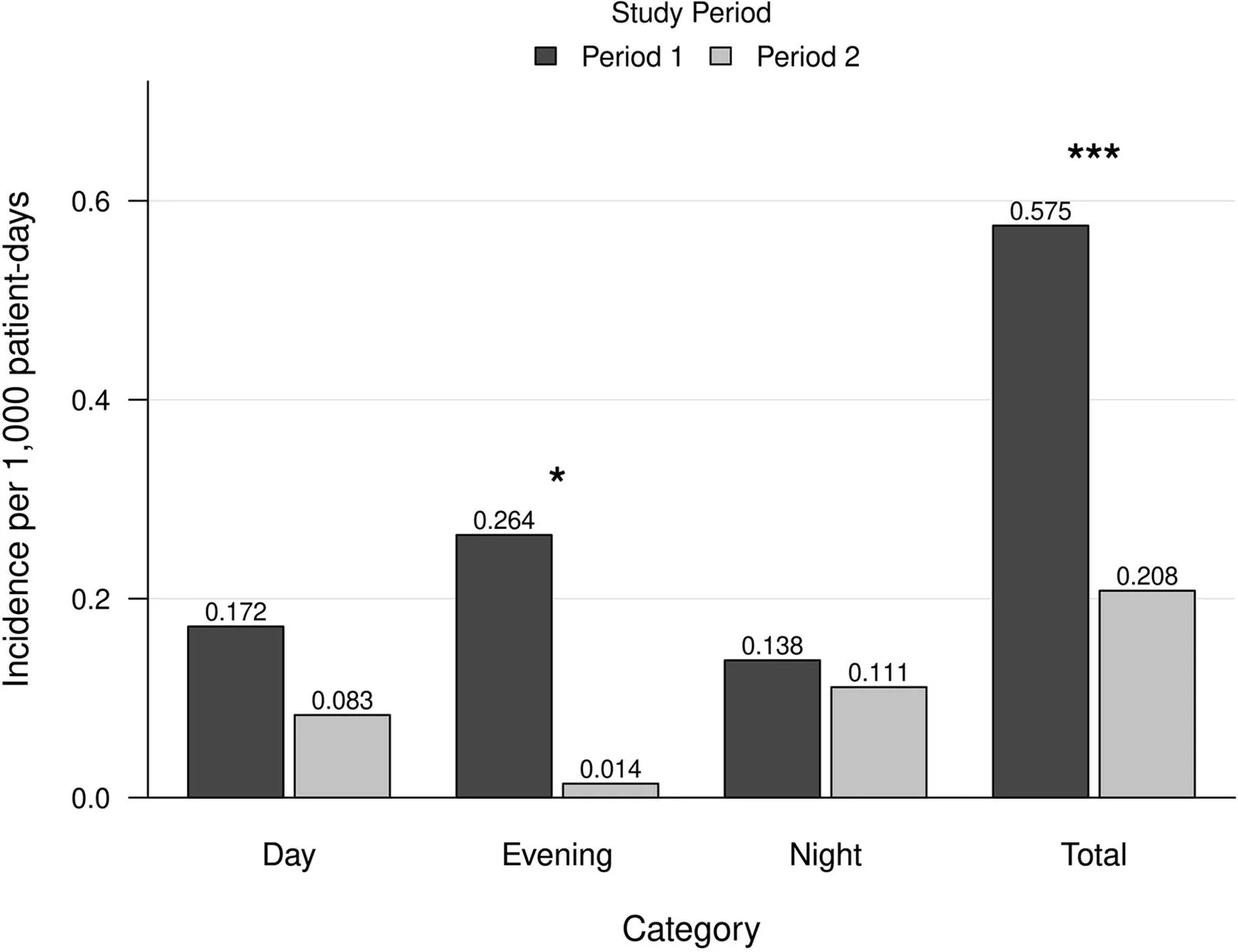
**Code Blue incidence rates by shift and total; with significant reduction of alarms in total and mainly during the Evening Shift**. *p = 0.000013, ^***^p = 0.00026. The IRR (95% CI) is for the Day Shift 0.48 (0.19–1.06), the Evening Shift 0.05 (0.001–0.32), the Night Shift 1.52 (0.32–7.69), and Total 0.36 (0.19–0.65).

### Demographics

No differences between periods were observed regarding age or sex. However, Code Blue alarms distribution across age cohorts was highly non-random (*p* < 0.00003), with significant overrepresentation of infants (<1 year; *z* = 3.29) and toddlers (1–6 years; *z* = 3.29) in the post-hoc analysis, accounting for 65.5% of all calls (38/65; (Fig. 3, Supplement 1, Table S1). While a significant reduction in alarms among infants and teenagers was observed during Period 2, alarm rates among toddlers remained largely unchanged (Fig. 3).

**Fig. 3:**
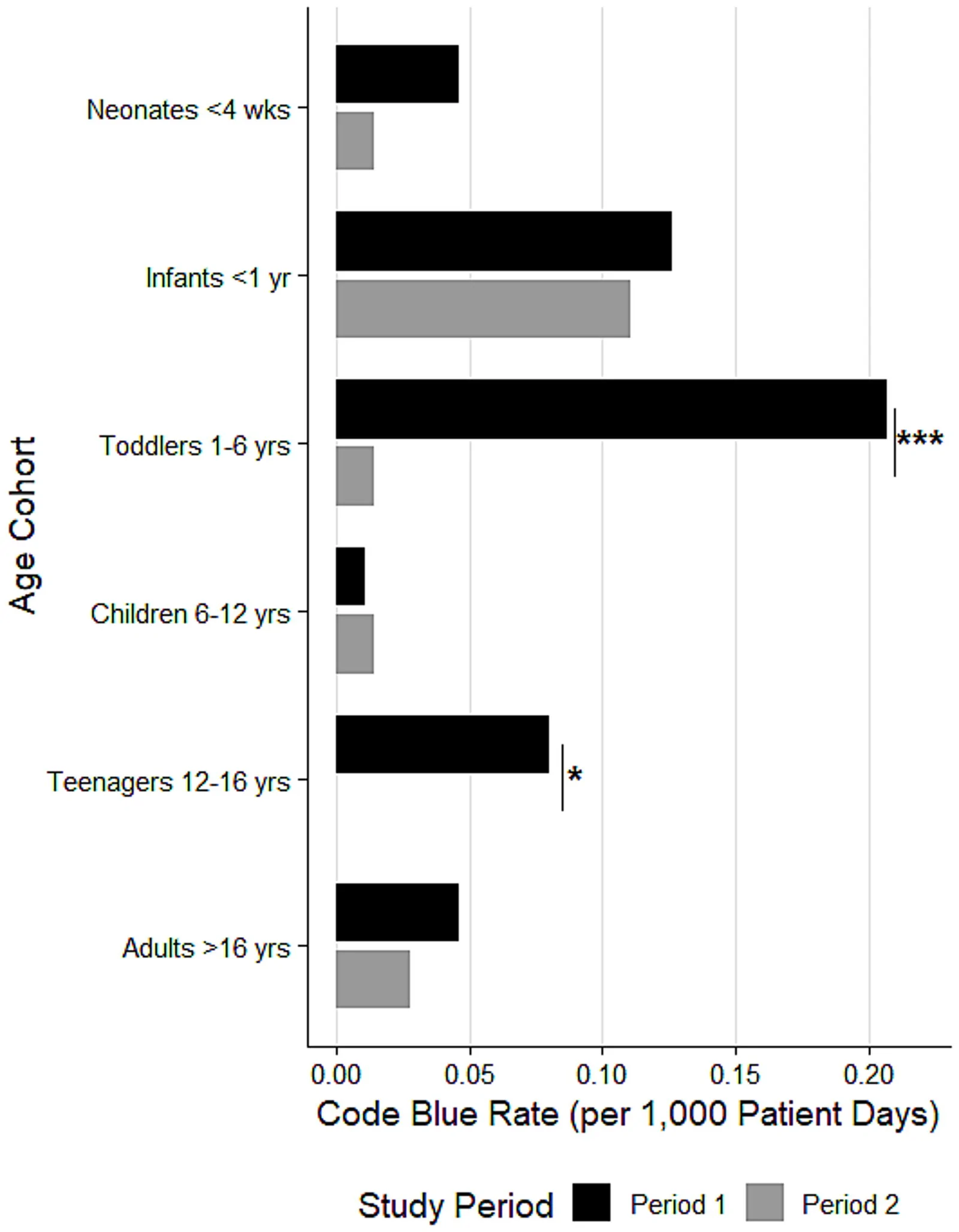
**Age cohorts; rate of alarms per 1000 patient-days**. wks: weeks; yr: year; yrs: years. ^***^p = 0.00017, *p = 0.015. 7 patients without documented age are not included in this figure.

### Temporal distribution and response times

Median response time was 2 min (IQR 1–2). Code Blue alarms were evenly distributed across the days of the week (*χ*^2^ = 4.03, *df* = 6, *p* = 0.67). The weighted comparison of activation frequency between the early week (Monday–Thursday; *n* = 36) and the extended weekend (Friday–Sunday; *n* = 29) showed no significant difference (so called “weekend effect”; *χ*^2^ = 0.082, *p* = 0.77). Details see Supplemental 1, Table S1.

Hourly analysis revealed the high peaks between 11–12 a.m. and 1–2 p.m. Outside of these intervals, the distribution was homogeneous (Supplemental 1, Table S2).

However, a distinct shift was observed regarding the diurnal distribution: While alarms occurred frequently across all shifts in Period 1, Period 2 showed a near-complete elimination of Code Blue calls during the evening shift (23 vs. 1 activation; *p*_adj_ = 0.017) and a halving of alarms during the day shift (Fig. 2). Alarm rates during the night shift remained largely stable.

### Clinical characteristics and interventions

The most frequent primary diagnoses were respiratory arrest (*n* = 17, 26.2%), and seizures (*n* = 11, 16.9%). Case-by-case analysis (Table 2) showed no significant shifts in diagnosis frequencies between period 1 and period 2 (*p* > 0.05). Although lower event counts were observed in specific categories such as anaphylaxis (4 vs. 1) and tracheal cannula dislocations (4 vs. 1), these did not reach statistical significance (*p* = 1.00) due to the low absolute event counts.

**Table 2:** Diagnoses, treatments, emergency locations, and admitting units.

	***n* total**	**%**	***z*-Score**	***n* Period 1**	***n* Period 2**
Total patients	**65**			**50**	**15**

Diagnosis group	*χ*^2^ = 56.7, df = 13, *p* < 0.001				
Respiratory arrest	17	26.2	5.95	11	6
Seizure	11	16.9	3.06	10	1
Respiratory other	6	9.2	0.65	5	1
Anaphylaxis	5	7.7	0.17	4	1
Cardiopulmonary arrest	5	7.7	0.17	4	1
Syncope	5	7.7	0.17	2	3
Tracheal cannula dislocation	5	7.7	0.17	4	1
Tetralogy of Fallot “crisis”	2	3.1	−1.27	2	0
Neurological other	2	3.1	−1.27	1	1
Pulmonary hypertension crisis	2	3.1	−1.27	2	0
Surgical other	2	3.1	−1.27	2	0
Hypoglycemia	1	1.5	−1.75	1	0
Cardiac other	1	1.5	−1.75	1	0
Psychiatry other	1	1.5	−1.75	1	0

Location of emergency	*χ*^2^ = 55.2, df = 12, *p* < 0.001				
*Infant Unit & Cardiology*	13	20	3.58	9	4
*Hemat-Oncology Unit*	12	18.5	3.13	10	2
*Respiratory & Infectious Diseases & General Pediatrics*	12	18.5	3.13	10	2
*Surgery Unit*	7	10.8	0.89	5	2
*Public area*	7	10.8	0.89	5	2
*Emergency Department*	6	9.2	0.45	6	0
*Neurology & Endocrinology & Nephrology*	2	3.1	−1.34	2	0
*Anesthesia Induction Room*	1	1.5	−1.79	1	0
*Day Clinic*	1	1.5	−1.79	0	1
*PICU*	1	1.5	−1.79	0	1
*Post-Anesthesia Care Unit*	1	1.5	−1.79	0	1
*Radiology*	1	1.5	−1.79	1	0
*Therapeutic Pool*	1	1.5	−1.79	1	0

**Admitting unit**					
*PICU*	31	47.7		24 (48%)	7 (46.7%)
*Treat and Release*	26	40.0		21 (42%)	5 (33.3%)
*ED (adults)*	5	7.7		3 (6%)	2 (13.3%)
*ED (pediatrics)*	3	4.6		2 (4%)	1 (6.7%)

*χ*^2^: Chi-quadrate; df: degree of freedom; *p*: *p*-value; BMV: Bag-Mask-Valve-Ventilation; CPR: Cardiopulmonary Resuscitation; PICU: Pediatric Intensive Care Unit; ED: Emergency Department. For statistical analysis, a Chi-square goodness-of-fit test was first conducted to determine uniformly distribution across the diagnoses as well as across the hospital units, revealing a significant non-uniform distribution (diseases: *χ*^2^ = 56.7, df = 13, *p* < 0.001; hospital units: *χ*^2^ = 55.2, df = 12, *p* < 0.001). Post-hoc analysis using standardized residuals identified two diagnoses (respiratory, seizure) and three clinical units as statistically significant hotspots (*z* > 1.96), indicating non-random clustering. The observed frequencies of individual diagnoses and emergency locations varied between Period 1 and Period 2, including marked changes in some categories. Given the small numbers in individual subgroups and the multiple-comparison issue, subgroup-specific *p*-values are not reported. Further breakdown by incidence rates per 1000 patient-days was deemed unnecessary.

Respiratory support was required in 33 cases (oxygen: 23.1%, BVM: 18.5%, intubation: 9.2%), while no respiratory support was needed in 49.2%. Additional interventions included medication administration (16.9%) and CPR (12.3%). Following Code Blue alarms 47.7% of patients were admitted to the pediatric intensive care unit (PICU; 47.7%), while 40% could be treated and released on scene.

### Spatial distribution of activations

The spatial distribution of emergencies demonstrated a highly significant, non-homogeneous pattern across hospital departments (*χ*^2^ = 55.2, *df* = 12, *p* < 0.001). Post-hoc analysis identified three wards as statistically significant hotspots (Table 1): the Infant & Cardiology Unit (*z* = 3.58), the Haemato-Oncology Unit (*z* = 3.13), and the Respiratory, Infectious Disease & General Pediatrics Unit (*z* = 3.13). These units contributed disproportionately to the total Code Blue alarm volume and thus, were trained particularly in their most detected emergencies: recognition of the acute ill infant with congenital heart defect (infant/cardiology ward), anaphylaxis (hemato-oncology ward), airway management and tracheal cannula dislocation (pneumology/general pediatric ward).

### Robustness and fragility analysis

In addition to acute Code Blue alarms, the frequency of formal MET consultations was analyzed to assess the baseline clinical workload. Only a slight decrease in consultations per 1000 patient-days was observed (1.23 vs. 0.94), but the difference did not reach statistical significance (*p* = 0.095), suggesting a relatively stable utilization of the team for MET consultations and providing no evidence of a substantial shift from Code Blue activations to MET consultations.

To address potential reporting bias, the fragility analysis revealed that 13 additional Code Blue events (totaling 28) would have had to remain undocumented in Period 2 to reach the tipping point of non-significance (*p* = 0.069). Given that 15 events were recorded, the event count in Period 2 would have had to be 80% higher than observed to nullify the statistical significance of the intervention.

As an additional post hoc sensitivity analysis, tertial 1/2021, which had the highest pre-intervention incidence (1.395 per 1000 patient-days), was excluded. The resulting pre-intervention incidence was 0.468 per 1000 patient-days, and the reduction compared in the post-intervention period remained statistically significant in a Poisson rate comparison (*p* = 0.007). Thus, the observed reduction was not solely attributable to the unusually high incidence in tertial 1/2021.

Despite the reduction in overall Code Blue activations, the relative proportion of patients requiring transfer to the intensive care unit remained unchanged between the study periods (48% vs. 47%; Table 1).

## Discussion

To our knowledge, this is the first study to examine the association between a ward- and disease-specific PBLS training concept and the frequency of pediatric Code Blue alarms. In this study, reorganization of PBLS training was associated with a significant reduction in Code Blue alarms in a tertiary children’s hospital. The observed effect remained stable across multiple robustness analyses. The analysis by tertiles also revealed a marked incidence peak in tertial 1/2021. This tertial occurred during the post-COVID19 phase and included approximately 2000 fewer patient-days than the other tertials on average. The CMI was also higher than in the subsequent pre-intervention tertiles of that year (1.40 vs. 1.26 and 1.25), suggesting some variation in patient case mix. Nevertheless, due to the small sample size random variation cannot be excluded, and the significant reduction remained evident after excluding tertial 1/2021.

Our novel approach differs from previously described PBLS and resuscitation training strategies in several important aspects: Existing concepts have primarily focused on repeated generic training formats, spaced learning, or low-dose high-frequency training approaches.^16^, ^17^, ^20^, ^21^ demonstrating that short and repeated refresher sessions improve cardiopulmonary resuscitation performance, skills, and compliance with guidelines. Our intervention expanded these concepts by integrating hotspot-specific clinical scenarios derived from actual emergencies occurring on individual wards. In summary, the training program was designed to enable ward teams to recognize deteriorating children at an early stage and to initiate the most important emergency measures independently during the first critical minutes before arrival of the emergency team.

Accordingly, we know that comparable emergency events continued to occur but could be managed at an early stage by the ward teams themselves without requiring Code Blue activation. For example, anaphylactic reactions continued to occur on the haemato-oncology ward, but fewer Code Blue activations were recorded after targeted training. These observations suggest that our intervention improved local competence and confidence in early emergency management.

As patient disposition and the relative rate of PICU admissions remained similar in both periods, these findings suggest that the emergency response system continued to identify and appropriately escalate patients requiring intensive care, despite the substantial reduction in Code Blue activations. Early recognition and intervention by ward staff may, meanwhile, have prevented some deteriorating patients from progressing to a Code Blue activation.

In our institution, the ward-based emergency equipment was deliberately streamlined toward core BLS measures. This decision reflected the organizational reality that our emergency team generally arrived within approximately two minutes. Under such conditions, complex advanced life support procedures including intubation, and preparation of emergency medications are difficult to perform effectively by ward personnel with limited exposure to these interventions. Reducing equipment complexity may therefore have facilitated faster and more reliable execution of essential stabilization measures until arrival of the emergency team.

Temporal analysis of Code Blue activations demonstrated no measurable weekend effect in our institution, consistent with the heterogeneous literature reporting conflicting findings on this topic.^22^, ^23^, ^24^, ^25^ However, a clustering of activations around midday was observed in our hospital. This period corresponds to lunch breaks and midday staff meetings affecting both nursing and medical personnel and likely represents a time of reduced availability of experienced staff on the wards.

Despite this, we did not observe increased alarm frequencies during nursing shift changes, as previously reported inconsistently in the literature.^25^, ^26^ Local organizational structures and staffing conditions may therefore play a greater role than temporal patterns alone. These factors should be considered important targets for future preventive strategies.

The predominance of affected infants in our cohort appears clinically plausible and in consistence with recent literature^27^: On the one hand, infants are particularly vulnerable to airway compromise because of their anatomy and respiratory pathophysiology. On the other hand, a substantial proportion of patients with congenital heart disease are treated within this age group, especially those in the interstage period, who are known to be at a particularly high risk for clinical deterioration. Although the small number of cases warrants cautious interpretation, cardiac-related emergencies decreased between the two study periods (Table 2). Nevertheless, infants remained the age group at highest risk for emergencies and therefore continue to represent a particularly vulnerable population. The combination of the potentially abrupt course of decompensation, awareness of its potentially fatal consequences, and the heightened sensitivity of ward staff to the specific risks of this population may therefore justify a persistently low threshold for Code Blue activation. Further analysis is warranted to determine whether more specific training scenarios can be implemented for this age group.

A notable aspect of our cohort was the occurrence of adult emergencies within the pediatric hospital setting. The possibility of adult emergencies requires specific consideration in the planning and organization of in-hospital emergency response systems in standalone children’s hospitals. Not only appropriate equipment and medications for adult patients must be available, but predominantly pediatric-trained staff must also be prepared to manage adult emergencies. In addition, clear pathways for further treatment and transfer of these patients need to be established.

Our study has several limitations. First, this was a single-center before–after study without a parallel control group – a design commonly encountered in studies of this kind and largely inherent to in hospital resuscitation research.

Second, although one ward was closed and integrated into another unit during Period 2, overall staffing levels, hospital capacity, patient CMI, and total patient-days remained largely unchanged throughout the study periods. Nevertheless, residual temporal confounding cannot be excluded, and other unmeasured factors independent of the training intervention may also have influenced the observed reduction in Code Blue activations. The intervention itself consisted of multiple simultaneous components, including training reorganization, implementation of hotspot-specific simulations, and restructuring of emergency equipment. The individual contribution of each component therefore cannot be isolated. It is possible that our observed effect reflects the cumulative impact of multiple organizational changes rather than a single intervention alone.

Third, the number of events was relatively small, which is a general difficulty in pediatric resuscitation research, and thus limiting statistical power. In addition, resuscitation structures, staffing models, and training concepts differ considerably between institutions, limiting comparability between hospitals, and thus making multicenter studies in this field challenging.

Finally, the observation period remains relatively short. It is well demonstrated that improvements in performance observed shortly after introduction of simulation-based interventions or CPR training may diminish over longer follow-up periods of 3–12 months.^21^, ^28^, ^29^ In our institution, however, the hotspot-specific training program has been maintained continuously rather than introduced as a temporary intervention, and we therefore expect a more sustained effect over time. In addition, we recently introduced low-fidelity, high-frequency simulation sessions^16^ in the PICU, with the aim of expanding this concept to the wards following positive evaluation. Nevertheless, longer follow-up analyses are required.

As we did not evaluate patient-centered outcomes such as mortality or neurological outcome, future studies should therefore examine whether hotspot-based ward training translates into measurable improvements in clinical outcomes in addition to reductions in Code Blue alarms or MET activations.

Despite these limitations, the present study demonstrates that systematic analysis of local emergency patterns can be used to redesign PBLS education in a clinically meaningful way. Identification of hotspot wards, vulnerable patient populations, and temporal peaks enabled targeted interventions tailored to the actual emergencies encountered by frontline teams. Other institutions may benefit from critically evaluating their own emergency response structures to implement focused preventive strategies adapted to their institutional setting.

## Declaration of generative AI and AI-assisted technologies in the manuscript preparation process

During the preparation of this work, the authors additional used “Elicit” and “openevidence” for literature research (Supplement 1, Table S4). After using this tool/service, the authors reviewed and edited the content as needed and took full responsibility for the content of the published article. Google Gemini (Imagen 3) was utilized solely for generating the individual icons within the graphical abstract, but not for the creation or structural design of the abstract itself.

## CRediT authorship contribution statement

**Volker Nils Umlauf:** Writing – original draft, Visualization, Validation, Supervision, Software, Resources, Project administration, Methodology, Investigation, Formal analysis, Data curation, Conceptualization. **Ruth Bieri:** Writing – review & editing, Resources, Project administration, Methodology, Conceptualization. **Daniela Studer:** Writing – review & editing, Project administration, Investigation, Conceptualization. **Thomas Riedel:** Writing – review & editing, Validation, Methodology.

## Funding

This research did not receive any specific grant from funding agencies in the public, commercial, or not-for-profit sectors.

## Declaration of competing interest

The authors declare that they have no conflicts of interest related to this work.

## Appendix A Supplementary material

Supplementary Data 1 Additional information on emergency team equipment, statistical methods and R packages, detailed analyses of Code Blue activations (Tables S1–S3), and documentation of AI-assisted literature searches (Table S4).
