# Supplementary material for: Hotspot-targeted life support training reduces code blue alarms in a tertiary children’s hospital: a single-center observational before–after study
Source: Resusc Plus. 2026 Oct 6;32:101520. doi: 10.1016/j.resplu.2026.101520 (PMC13695375; doi:10.1016/j.resplu.2026.101520)
Supplement: Supplementary file 1 [file mmc1.docx]

**Supplemental 1**

*Equipment*

Pediatric Medical Emergency Team Children’s Hospital Bern

- Monitoring: Zoll X-Series monitor/defibrillator (with 12-lead ECG)
- Oxygen bag with a 2 L oxygen cylinder
- Suction unit: Laerdal Compact Suction Unit LCSU 4
- emergency medical kit bag

*R packages*

- Data cleaning, transformation, and descriptive analysis: *tidyverse* ecosystem
- data import: *readxl* (v1.4.3) package
- data manipulation: *dplyr* (v1.1.4) and *tidyr* (v1.3.1)
- cross-tabulation and header cleaning: *janitor* (v2.2.0)

*Statistical Tests*

Continuous variables were expressed as medians with interquartile ranges (IQR) and compared between periods using the Wilcoxon rank-sum test. Categorical variables were presented as absolute frequencies and percentages; inter-group comparisons (period 1 vs. period 2) were performed using Fisher’s exact test.

To account for the different lengths of the observation periods (period 1: 28 months; period 2: 24 months), Code Blue incidence was compared using a Poisson test to calculate the Incidence Rate Ratio (IRR) and 95% confidence intervals (CI). To evaluate the robustness of the primary results, a sensitivity analysis (stress testing; tipping point analysis) was performed to determine the impact of potential underreporting (missing data) in Period 2 on statistical significance.

To evaluate the distribution of categorical variables across the total cohort (e.g., age categories, weekdays, diseases, and activation locations), a Chi-square goodness-of-fit test was employed. Following a significant result, post-hoc analysis was performed by calculating standardized Pearson residuals; a standardized residual (z-score) > 1.96 was defined as a statistically significant deviation.

To analyze the "weekend effect," weekdays were grouped into "early week" (Monday–Thursday) and "extended weekend" (Friday–Sunday). A weighted Chi-square goodness-of-fit test was applied, with expected frequencies adjusted to the 4:3 ratio to account for the unequal number of days.

The temporal distribution of Code Blue calls was further analyzed by nursing shifts: Day Shift (07:00–15:00), Evening Shift (15:00–23:00), and Night Shift (23:00–07:00). A global Fisher’s exact test was used to assess shifts in the overall distribution between periods. Following a significant global result, post-hoc pairwise Fisher’s exact tests were conducted for each shift. To maintain a family-wise error rate of α = 0.05 and account for multiple comparisons, Bonferroni correction was applied (padj = p * 3). Statistical significance for all tests was defined as a two-tailed p < 0.05 (or padj < 0.05 where applicable).

|  | **Total** |  | **Period 1** | **Period 2** |  |
| --- | --- | --- | --- | --- | --- |
| **Period** |  |  | 01/2021 – 4/2023 | 1/2024 – 12/2025 |  |
| months | 52 |  | 28 | 24 |  |
| patient days |  |  | 86966 | 72141 |  |
| CMI (mean) |  |  | 1.41 | 1.44 |  |
|  |  |  |  |  |  |
| **Code Blue Calls** *(n)* | 65 |  | 50 | 15 | *p* = 0.00015^d^  *RR* 2.86; 95% *CI* 1.58–5.48 |
| *incidents per month* | 1.25 |  | 1.79 | 0.63 |  |
| *incidents per 1000 days* |  |  | 0.575 | 0.208 | p = 0.00025 |
| ***MET consultations*** |  |  | 107 | 68 | p = 0.058  RR 0.74; 95% CI 0.52–1.01 |
| *incidents per month* |  |  | 3.82 | 2.83 |  |
| *incidents per 1000 days* |  |  | 1.230 | 0.943 | p = 0.095, RR 1.31; 95% CI 0.95–1.80 |
|  | | | | | |
| **Day of Week** | p = 0.67, df = 6, *χ*^2^ = 4.03 | |  |  |  |
| *Monday* | 7 |  | 6 | 1 |  |
| *Tuesday* | 6 |  | 5 | 1 |  |
| *Wednesday* | 12 |  | 8 | 4 |  |
| *Thursday* | 11 |  | 6 | 5 |  |
| *Friday* | 11 |  | 9 | 2 |  |
| *Saturday* | 11 |  | 10 | 1 |  |
| *Sunday* | 7 |  | 6 | 1 |  |
| *Week Mon–Thu* | 36 | p = 0.77^e^, *χ*^2^ = 0.082 | 25 | 11 |  |
| *Weekend Fri–Sun* | 29 |  | 25 | 4 |  |
|  | | | | | |
| **Time of Day** |  |  |  |  |  |
| **hours** | *χ*^2^ = 41,54, df = 24, p = 0.0145 | | | | |
| *11:00–12:00 am* | 7 | z = 2.15 |  |  |  |
| *1:00–2:00 pm* | 6 | z = 2.79 |  |  |  |
| **shifts** | *χ*^2^ = 12.59, df = 2, p = 0.0019 | |  |  |  |
|  | 65 |  | 50 | 15 | p = 0.0001899 |
| *incidents per 1000 days* |  |  | 0.575 | 0.208 | p = 0.000255  RR 2.76; 95% CI 1.53–5.3 |
| *Day Shift* | 32 |  | 23 | 9 | p = 0.0408, p adj = 0.1223 |
| *incidents per 1000 days* |  |  | 0.172 | 0.083 | p = 0.05  RR 2.12; 95% CI 0.94–5.2 |
| *Evening Shift* | 24 |  | 23 | 1 | p = 0.000037, p adj = 0.017 |
| *incidents per 1000 days* |  |  | 0.264 | 0.014 | p = 0.000013  RR 19.07; 95% CI 3.1–785.75 |
| *Night Shift* | 9 |  | 4 | 5 | p = 0.5715, p adj = 1.0 |
| *incidents per 1000 days* |  |  | 0.138 | 0.111 | p = 0.74  RR 0.66; 95% CI 0.13–3.08 |
|  | | | | | |
| ***Age (years)****(median, IQR)* | 1.80 [0.53–5.88] |  | 2.05 [0.59–6.11] | 0.84 [0.26–4.62] | p = 0.33^a^ |
| Age cohorts | *χ*^2^ = 28.48, df = 5, *p* = 0,00003 | | | | |
| ≤4 wks | 5 | -1.64^e^ | 4 | 1 |  |
| <1 yr | 19 | 3.29^e^ | 11 | 8 |  |
| 1–6 yrs | 19 | 3.29^e^ | 18 | 1 |  |
| 7–12 yrs | 2 | -2.70^e^ | 1 | 1 |  |
| 13–16 yrs | 7 | -0.94^e^ | 7 | 0 |  |
| >16 yrs | 6 | -1.29^e^ | 4 | 2 |  |
| NA | 7 |  | 5 | 2 |  |
|  |  |  |  |  |  |
| **Sex** |  |  |  |  | *p* = 0.25^b^ |
| *F* | 32 (49 %) |  | 26 (52 %) | 6 (40 %) |  |
| *M* | 28 (43 %) |  | 19 (38 %) | 9 (60 %) |  |
| *Unknown* | 5 (8 %) |  | 5 (10 %) | 0 (0 %) |  |
|  |  |  |  |  |  |
| **Time** *(median, IQR)* |  |  |  |  |  |
| *Response Time* | 2 [1–2] |  | 2 [1–2] | 2 [1–2.75] | *W* = 219.5, *p* = 0.52^c^ |
| *Time on Scene* | 15 [8.75–25] |  | 16 [9–25] | 9 [6.5–17] | *W* = 323.5, *p* = 0.14 |
| *Total time* | 20 [11–31] |  | 20.5 [11.5–31.75] | 17.5 [11.75–24.5] | *W* = 286.5, *p* = 0.47 |

**Supplement Table S1:** All subgroup analyses refer to Code Blue alarms. ^a^ Wilcoxon rank-sum test; ^b^ Fisher test; ^c^ Wilcoxon rank-sum test with continuity correction; ^d^ Poisson test; ^e^ Chi-square goodness-of-fit test; standard residuals calculation (Pearson residuals); RR: rate ratio; CI: confidence interval; IQR: Interquartile Range; CMI: Case Mix Index; F: female; M: male

The distribution of Code Blue alarms across age cohorts was highly non-random (p < 0.00003, *χ*^2^ goodness-of-fit test). Post-hoc analysis using standardized residuals confirmed a significant overrepresentation of infants (< 1 year; *z* = 3.29) and toddlers (1–6 years; z = 3.29), who together accounted for 65.5% of all Code Blue calls (n=38/58). Conversely, children aged 6–12 years were significantly less likely to require Code Blue emergency team intervention (*z* = -2.70).

| **hour** | **n** | **%** | **expected** | **Residual** | **Interpretation** |
| --- | --- | --- | --- | --- | --- |
| 0–1 | 1 | 1.5 | 2.6 | -1.0127394 | Normal Variation |
| 1–2 | 2 | 3.1 | 2.6 | -0.3797773 | Normal Variation |
| 2–3 | 0 | 0.0 | 2.6 | -1.6457015 | Normal Variation |
| 3–4 | 0 | 0.0 | 2.6 | -1.6457015 | Normal Variation |
| 4–5 | 0 | 0.0 | 2.6 | -1.6457015 | Normal Variation |
| 5–6 | 1 | 1.5 | 2.6 | -1.0127394 | Normal Variation |
| 6–7 | 0 | 0.0 | 2.6 | -1.6457015 | Normal Variation |
| 7–8 | 4 | 6.2 | 2.6 | 0.8861469 | Normal Variation |
| 8–9 | 2 | 3.1 | 2.6 | -0.3797773 | Normal Variation |
| 9–10 | 5 | 7.7 | 2.6 | 1.5191091 | Normal Variation |
| 10–11 | 3 | 4.6 | 2.6 | 0.2531848 | Normal Variation |
| 11–12 | 6 | 9.2 | 2.6 | 2.1520712 | High Peak (Hotspot) |
| 12–13 | 5 | 7.7 | 2.6 | 1.5191091 | Normal Variation |
| 13–14 | 7 | 10.8 | 2.6 | 2.7850333 | High Peak (Hotspot) |
| 14–15 | 0 | 0.0 | 2.6 | -1.6457015 | Normal Variation |
| 15–16 | 5 | 7.7 | 2.6 | 1.5191091 | Normal Variation |
| 16–17 | 3 | 4.6 | 2.6 | 0.2531848 | Normal Variation |
| 17–18 | 4 | 6.2 | 2.6 | 0.8861469 | Normal Variation |
| 18–19 | 3 | 4.6 | 2.6 | 0.2531848 | Normal Variation |
| 19–20 | 3 | 4.6 | 2.6 | 0.2531848 | Normal Variation |
| 20–21 | 3 | 4.6 | 2.6 | 0.2531848 | Normal Variation |
| 21–22 | 1 | 1.5 | 2.6 | -1.0127394 | Normal Variation |
| 22–23 | 2 | 3.1 | 2.6 | -0.3797773 | Normal Variation |
| 23–24 | 0 | 0.0 | 2.6 | -1.6457015 | Normal Variation |
| NA | 5 | 7.7 | 2.6 | 1.5191091 | Normal Variation |

**Supplement Table S2:** Distribution of Code Blue alarms across the 24-hour day cycle. Hours >12: times p.m.

A Chi-square goodness-of-fit test was first conducted to determine uniformly distribution across 24 hours, revealing a significant non-uniform distribution (*χ*^2^ = 41.54, df = 24, p = 0.015). Post-hoc analysis using standardized Pearson residuals identified the hours 11–12 and 13 – 14 (= 1–2 p.m.) as the largest positive deviations from the expected frequency.

|  | **n total** | **%** |
| --- | --- | --- |
| **Treatment** |  |  |
| Respiratory support |  |  |
| *none* | 32 | 49.2 |
| *oxygen* | 15 | 23.1 |
| *BMV* | 12 | 18.5 |
| *intubation* | 6 | 9.2 |
|  |  |  |
| other |  |  |
| *none* | 46 | 70.8 |
| *medication* | 11 | 16.9 |
| *CPR* | 8 | 12.3 |
|  |  |  |

**Supplement Table S3:** Total treatments

| **AI model** | **prompts** |
| --- | --- |
| **openevidence** | - Is there evidence for time patterns and different causes of medical emergency calls (rapid response calls, code blue calls) in pediatric wards (not ICU)?   - Is there literature on typical hot spots in children's hospitals?   - Are there more data about the time or weekdays occurrence of pediatric emergency?   - Has the implementation of rapid response teams (medical emergency teams) shown reduction in mortality in children's hospitals? - Is there evidence for increased rapid response system activations during shift changes and break periods and on weekends? - Is there evidence on how many patients (children) from medical emergency team activations / rapid response team activations have to transmitted - Is there evidence for structured training programs on life support can reduce medical emergency team activations in children's hospitals?   - identify and summarize any high-quality studies or systematic reviews that specifically measure the effect of structured life support training on the actual rates of medical emergency team activations in pediatric hospitals - Is there evidence for improvement in patient safety by reorganizing basic life support training in hospitals?   - Is there evidence that reorganized bls training has an influence on the outcome of patients and pediatric patients?   - Is there evidence of the influence of reorganized bls training on code blue alarms or medical emergency team activations?   - Is there evidence that less resuscitation material is superior to complex resuscitation trolleys in non ICU areas? |
| **elicit** | - Give me an overview on pediatric medical emergency teams (rapid response team) and the correlation of time of activation and weekdays or day-hours - Is there an association between time or weekday between number of code blue calls or rapid response team (medical emergency team) activations and time or day depending mortality in children's hospitals? |

**Supplement Table S4:** AI prompts with in-conservation follow-up prompts
